# Editorial Notes

**Published:** 1882-03

**Authors:** 


### EDITORIAL NOTES.

The suit brought against Messrs. Parke, Davis & Co. by an English firm to prevent their use of the word "Tonga" as a title for a combination of barks used in Fiji as a specific for neuralgia, and introduced into this country by P., D. & Co., has terminated by the withdrawal of the suit by the London firm.

We call attention to the advertisement of "Dyspepsin," a combination of pepsin, pancratine and malt. The commendation which this preparation has received from the most eminent medical men of Toronto, and their extensive employment of it, makes it well worthy of a trial. We have received a quantity for trial, and will report our experience with it.

In imitation of the plan adopted by the London University, the Illinois Wesleyan University at Bloomington, Ill., has had for nearly ten years courses of study open to non-resident students, with non-resident examinations. These courses lead to the degree of Ph. B., M. A., and Ph. D. Catalogues containing full information will be sent on application to Prof. C. M. Moss.

One of the pleasant incidents, connected with Commencement-Day at the University of Buffalo, was the gift of Dr. White's valuable medical library to the Medical College by Mr. James P. White, who manifests in this generous donation the earnest interest of the family in the welfare of the college for which Dr. White so long and successfully labored. We are gratified to witness this disposition of the library, and also to commend the spirit which actuated the donor.

The firm of Lindsay & Blakiston, which for the last forty years has had a corporate existence, is dissolved by the retirement of Mr. Lindsay. The business is continued under the firm name of P. Blakiston, Son & Co. May the new firm retain the confidence and esteem which for nearly half a century was most cordially given by the medical profession and most thoroughly deserved by Lindsay & Blakiston, whose name was as the name of an old friend to the majority of medical men.

The seventy-sixth annual meeting of the State Medical Society, which was held in Albany early in February, was eminently a successful one. About thirty papers were presented and discussed, and besides the scientific portion of the work, some important measures were introduced relating to the profession as a whole, one of which we notice elsewhere. The volume of "Transactions" will of course contain the best communications in full, and they can then be noticed more at length.

At the Buffalo State Asylum for the Insane, under the able management of Dr. Judson B. Andrews, there are now confined 192 insane persons—over 300 having been received into this institution since it was opened for the reception of patients. The work has increased to such an extent that the second assistant physician has become necessary, and Dr. Lloyd S. Crego has been appointed to the position. This is an admirable appointment, and shows wise judgment upon the part of the Superintendent. The Buffalo Asylum, in a brief period, has secured an enviable reputation in the care and treatment of the insane.

PROF. FLINT favored Buffalo with a brief visit to attend the late commencement exercises of the Buffalo Medical College, and in the very able address which he delivered to the Alumni he very happily referred to his residence here from 1836 to 1859, and to the labors of the late Dr. White, which he shared, in the organization of the Medical College. The address was one of the most happy efforts of the eminent Professor, in which he brought to light the experiences of his early life in the profession which he now adorns. He paid a just tribute to the worth of his associate, Dr. White, and the undaunted will and tireless energy through which his great success was won. The speaker gave a just estimate of the American profession. We hope to be permitted to publish this very interesting address in a future number of the JOURNAL.

"NO ODORS SWEET PROCLAIM THE SPOT."—The sanitary, or rather the unsanitary, condition of East Buffalo has again been brought to public notice by the prevalence of sickness and the large number of deaths reported from that vicinity. This condition of things is by many attributed to the presence of two barns in which a considerable number of cows are confined, and also to the proximity of a fat-boiling establishment. That these places are public nuisances, in that they render a residence near them unpleasant, is beyond a question; but that they directly injure the health of the neighborhood we greatly doubt. A far more reasonable explanation of the peculiar prevalence of "typhoid" is the condition of the public wells. That many of these were unfit for use was shown in a report published by Dr. Davidson as long ago as 1879. Again, by order of the Board of Health, the same chemist has recently examined the water of the wells in the vicinity. His report shows at least one good and sufficient reason for the unhealthfulness of this locality. These contaminated and poisoned wells should be at once closed; there is more danger from one such well than from a dozen cattle barns. There seems to be at this time a disposition on the part of the civic authorities to support the Board of Health in their efforts to correct the evils so bitterly complained of by the residents of this section of the city.

